# Chronic implantable flexible serpentine probe reveals impaired spatial coding of place cells in epilepsy

**DOI:** 10.1093/nsr/nwae402

**Published:** 2024-11-15

**Authors:** Yu Wang, Meiqi Han, Zhaojie Xu, Shiya Lv, Gucheng Yang, Fan Mo, Luyi Jing, Qianli Jia, Yiming Duan, Wei Xu, Peiyao Jiao, Yaoyao Liu, Jin Shan, Ming Li, Mixia Wang, Jinping Luo, Yilin Song, Juntao Liu, Yirong Wu, Xinxia Cai

**Affiliations:** State Key Laboratory of Transducer Technology, Aerospace Information Research Institute, Chinese Academy of Sciences, Beijing 100190, China; School of Electronic, Electrical and Communication Engineering, University of Chinese Academy of Sciences, Beijing 100049, China; State Key Laboratory of Transducer Technology, Aerospace Information Research Institute, Chinese Academy of Sciences, Beijing 100190, China; School of Electronic, Electrical and Communication Engineering, University of Chinese Academy of Sciences, Beijing 100049, China; State Key Laboratory of Transducer Technology, Aerospace Information Research Institute, Chinese Academy of Sciences, Beijing 100190, China; School of Electronic, Electrical and Communication Engineering, University of Chinese Academy of Sciences, Beijing 100049, China; State Key Laboratory of Transducer Technology, Aerospace Information Research Institute, Chinese Academy of Sciences, Beijing 100190, China; School of Electronic, Electrical and Communication Engineering, University of Chinese Academy of Sciences, Beijing 100049, China; State Key Laboratory of Transducer Technology, Aerospace Information Research Institute, Chinese Academy of Sciences, Beijing 100190, China; School of Electronic, Electrical and Communication Engineering, University of Chinese Academy of Sciences, Beijing 100049, China; State Key Laboratory of Transducer Technology, Aerospace Information Research Institute, Chinese Academy of Sciences, Beijing 100190, China; School of Electronic, Electrical and Communication Engineering, University of Chinese Academy of Sciences, Beijing 100049, China; State Key Laboratory of Transducer Technology, Aerospace Information Research Institute, Chinese Academy of Sciences, Beijing 100190, China; School of Electronic, Electrical and Communication Engineering, University of Chinese Academy of Sciences, Beijing 100049, China; State Key Laboratory of Transducer Technology, Aerospace Information Research Institute, Chinese Academy of Sciences, Beijing 100190, China; School of Electronic, Electrical and Communication Engineering, University of Chinese Academy of Sciences, Beijing 100049, China; State Key Laboratory of Transducer Technology, Aerospace Information Research Institute, Chinese Academy of Sciences, Beijing 100190, China; School of Electronic, Electrical and Communication Engineering, University of Chinese Academy of Sciences, Beijing 100049, China; State Key Laboratory of Transducer Technology, Aerospace Information Research Institute, Chinese Academy of Sciences, Beijing 100190, China; School of Electronic, Electrical and Communication Engineering, University of Chinese Academy of Sciences, Beijing 100049, China; State Key Laboratory of Transducer Technology, Aerospace Information Research Institute, Chinese Academy of Sciences, Beijing 100190, China; School of Electronic, Electrical and Communication Engineering, University of Chinese Academy of Sciences, Beijing 100049, China; State Key Laboratory of Transducer Technology, Aerospace Information Research Institute, Chinese Academy of Sciences, Beijing 100190, China; School of Electronic, Electrical and Communication Engineering, University of Chinese Academy of Sciences, Beijing 100049, China; State Key Laboratory of Transducer Technology, Aerospace Information Research Institute, Chinese Academy of Sciences, Beijing 100190, China; School of Electronic, Electrical and Communication Engineering, University of Chinese Academy of Sciences, Beijing 100049, China; State Key Laboratory of Transducer Technology, Aerospace Information Research Institute, Chinese Academy of Sciences, Beijing 100190, China; School of Electronic, Electrical and Communication Engineering, University of Chinese Academy of Sciences, Beijing 100049, China; State Key Laboratory of Transducer Technology, Aerospace Information Research Institute, Chinese Academy of Sciences, Beijing 100190, China; School of Electronic, Electrical and Communication Engineering, University of Chinese Academy of Sciences, Beijing 100049, China; State Key Laboratory of Transducer Technology, Aerospace Information Research Institute, Chinese Academy of Sciences, Beijing 100190, China; School of Electronic, Electrical and Communication Engineering, University of Chinese Academy of Sciences, Beijing 100049, China; State Key Laboratory of Transducer Technology, Aerospace Information Research Institute, Chinese Academy of Sciences, Beijing 100190, China; School of Electronic, Electrical and Communication Engineering, University of Chinese Academy of Sciences, Beijing 100049, China; State Key Laboratory of Transducer Technology, Aerospace Information Research Institute, Chinese Academy of Sciences, Beijing 100190, China; School of Electronic, Electrical and Communication Engineering, University of Chinese Academy of Sciences, Beijing 100049, China; State Key Laboratory of Transducer Technology, Aerospace Information Research Institute, Chinese Academy of Sciences, Beijing 100190, China; School of Electronic, Electrical and Communication Engineering, University of Chinese Academy of Sciences, Beijing 100049, China; State Key Laboratory of Transducer Technology, Aerospace Information Research Institute, Chinese Academy of Sciences, Beijing 100190, China; School of Electronic, Electrical and Communication Engineering, University of Chinese Academy of Sciences, Beijing 100049, China

**Keywords:** flexible probe, epilepsy, chronic neural recording, place cell, biocompatible surface

## Abstract

The development of minimally invasive and reliable electrode probes for neural signal recording is crucial for advancing neuroscience and treating major brain disorders. Flexible neural probes offer superior long-term recording capabilities over traditional rigid probes. This study introduces a parylene-based serpentine electrode probe for stable, long-term neural monitoring. Inspired by the flexibility and morphology of snakes, the serpentine design of the probe ensures stable anchorage within the brain tissue during subject movement. The probe features a hydrophilic surface and is combined with a biodegradable silk fibroin–polyethylene glycol coating, significantly enhancing biocompatibility and mitigating inflammatory responses. *In vivo* experiments demonstrate that these probes enable stable, high-quality neural recordings for >8 months. The probes are also used to investigate the neural bases of epilepsy-induced cognitive deficits. By analysing place-cell dynamics in mice pre- and post-epileptic events, we identified the correlation between impaired spatial encoding and the observed cognitive deficits in epileptic mice. This study highlights the potential of our flexible probes in neurological research and medical applications.

## Teaser

Over 8 months, our flexible serpentine neural probe provides stable recordings with minimal inflammation, revealing that abnormal place-cell encoding underlies epilepsy-induced cognitive deficits.

## INTRODUCTION

Epilepsy ranks among the most prevalent neurological conditions [1], characterized primarily by the occurrence of epileptic seizures. Beyond seizures, individuals with epilepsy often develop cognitive impairments [2], significantly affecting their quality of life. The dominant pathophysiological perspective suggests that seizures and epileptiform discharges directly impair the neural networks that are essential for normal cognitive functions [3]. Within the temporal lobe, the hippocampus serves as a pivotal brain area for encoding cognitive maps [4,5], with place cells being the key cell type that is involved in spatial cognition. These cells, which are responsible for encoding spatial information, demonstrate high discharge rates in specific environmental regions and low responses elsewhere [6]. However, under the influence of temporal lobe epilepsy (TLE), hippocampal damage occurs, which broadly impairs cognitive functions. Numerous studies have indicated that spatial memory is compromised during both the latency and interictal phases following early seizures [7], as well as in status epilepticus (SE) that is treated with pilocarpine [8]. The onset of these cognitive deficits may be linked to alterations in hippocampal activity, including disrupted plasticity mechanisms [9], a reduced proportion of place cells [10] and diminished theta oscillation power [11,12]. Yet, the precise cellular and neural circuitry mechanisms that underlie cognitive deficits in TLE remain elusive. An investigation of the changes in place-cell coding at the cellular level to elucidate cognitive deficit mechanisms can provide a theoretical basis for novel therapeutic approaches.

To more effectively monitor and diagnose the impacts of epilepsy on the hippocampus, the use of microelectrode arrays (MEAs) has been increasingly adopted [11,13]. Implanted MEAs can be positioned within deep brain regions to capture high-resolution neural information, enabling precise detection of individual neuronal activity and pathological changes. MEAs serve as the front end for signal acquisition and stimulation control, making the development of safe, durable and high-quality neural microelectrodes a critical direction in the entire brain–machine interface technology spectrum. However, traditional rigid electrode probes, often based on materials such as silicon with a modulus of elasticity ranging from 50 to 200 GPa, exhibit mechanical mismatch compared with brain tissue, which has a modulus that ranges from 3.15 to 15 kPa [14]. This disparity leads to the identification of electrode probes as foreign bodies, which can be prone to the elicitation of a foreign body response (FBR) in the brain [15]. The brain FBR triggers reactive gliosis that involves microglia and astrocytes, which typically results in the formation of a glial scar that encapsulates the MEAs [16]. This glial encapsulation gradually insulates the probes over time, leading to a significant decline in signal quality and, in some cases, rendering the signals undetectable. As a result, flexible electrode probes have emerged as a significant research focus, showing potential in minimizing brain-tissue damage and enhancing signal acquisition from deeper brain regions. Various surface modifications, including natural and synthetic coatings, hydrogel materials and morphological designs, have shown promising results in promoting neuronal adhesion, reducing glial proliferation and extending electrode durability [15]. Hydrogel electrodes with polyrotaxane structures as cross-linking agents have successfully recorded local field potentials (LFPs) over 8 weeks [17]. Nanoelectronic thread (NET) electrodes have diminished neuroglial scarring and maintained stable functionality for months [18,19]. By combining 3D-sharpened silicon shuttles with flexible polymer probes, it is possible to avoid dura mater incision and reduce postoperative edema, enabling stable recordings for >95 days [20]. Flexible graphene neural probes have demonstrated long-term functionality, maintaining performance for 10 weeks in rat models of absence epilepsy [21]. Soft polymeric materials such as polyimide (PI) [22], parylene [23], SU-8 [24] and liquid crystal polymer (LCP) [25], which have elastic moduli that range from 1 to 10 GPa, have become widely used as electrode materials due to their flexibility and excellent biocompatibility. Furthermore, polydimethylsiloxane (PDMS) [26], which has a lower modulus (1 MPa), has emerged as one of the softest substrates. However, its high water vapor transmission rate and limited precision in the patterning process continue to pose challenges. As the softness of the material increases, its processing difficulty also rises. To achieve precise micro- and nano-fabrication and enhance electrode density, parylene, which is both flexible and compatible with microelectromechanical systems (MEMS) technology, has emerged as an ideal substrate for electrodes.

To acquire stable *in vivo* neural data and investigate the impact of epilepsy on the functionality of place cells, we fabricated parylene-based flexible microelectrode array probes that were shaped like serpent spears. Following surface hydrophilization and modification with a silk fibroin (SF)–polyethylene glycol (PEG) coating, the biocompatible and soft serpent spear probes enabled long-term stable monitoring for ≤8 months *in vivo*. We conducted tracking and recording of the place cells in mice before and after epilepsy onset, and examined changes in their spatial encoding. These results revealed the connection between impairments in place-cell encoding and cognitive deficits induced by epilepsy.

## RESULTS

### Design and fabrication of flexible serpentine probe

**Figure 1.**
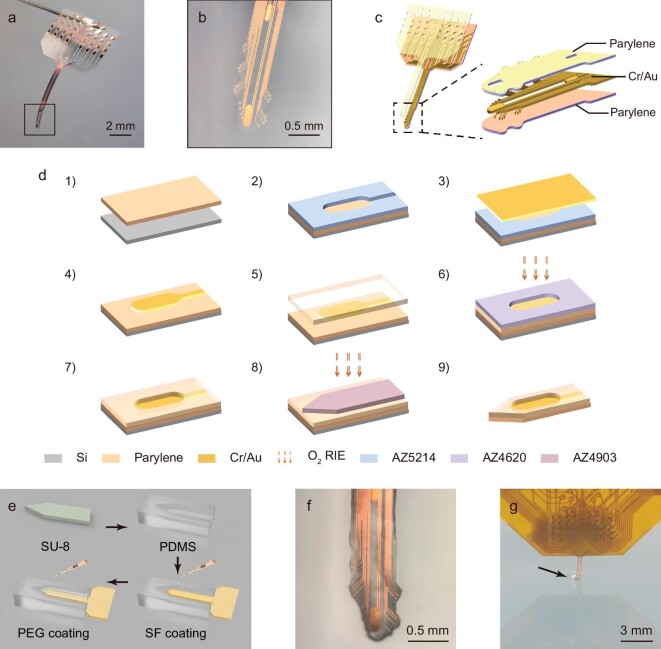
Morphology and fabrication of flexible serpentine probes. (a) Complete image of the flexible probe. (b) Details of the probe tip shown in the black rectangle of image (a). (c) Schematic diagram of the probe structure. (d) Fabrication process of the flexible probe. (1) Deposit a parylene film on a silicon wafer. (2) Use photolithography to pattern the metal layer. (3)–(4) Form the Cr/Au conductive layer by the lift-off process. (5) Deposit a parylene insulating layer. (6)–(7) Use photolithography to pattern the exposed regions and etch the film with oxygen plasma. (8) Photolithography defines the outline pattern of the probe, followed by etching. (9) Obtain the independent flexible probe. (e) PDMS mold manufacturing and temporary reinforcement scheme. (f) Photograph of a flexible probe reinforced with SF-PEG. (g) Flexible probe implanted in 0.6% agarose gel.

To ensure long-term stability and reliability in detection, this study introduces a parylene-based flexible electrode probe (Fig. 1a). The design of the frontal section of the probe (Fig. 1b) is inspired by the serpentine shapes that are found in nature—particularly the flexible and undulating movement of snakes. The tip features a wavy, curved structure that resembles a snake's body, which allows the probe to engage firmly with the surrounding brain tissue upon insertion. This biomimetic design enables the probe to anchor more effectively, minimizing relative movement during motion and ensuring stable and consistent long-term neural recordings. Furthermore, the rounded tip minimizes insertion-related damage [27]. It has been observed that, over time, electrode sites that are located on the periphery of the probe exhibit higher levels of activity compared with those at the center, resulting in superior signal quality [28,29]. The flexible serpentine probe includes a total of 32 recording electrode sites in addition to four reference/stimulation sites, strategically positioned at the wavy edges to facilitate consistent, long-term neural information detection. The electrical stimulation sites exhibit a charge storage capacity (CSC) as high as 19.69 ± 0.42 mC/cm², enabling effective neural activation. Although electrical stimulation modulation was not performed in this study, this high CSC will be a focus of future research.

Figure 1c illustrates the serpentine probe structure, which utilizes parylene-C for the substrate and insulating layer, and Cr/Au for the conductive layer. In this research, flexible serpentine probes were prepared by using the microelectromechanical systems (MEMS) process (Fig. 1d). The mechanical properties of the probes were evaluated by using PeakForce quantitative Nanoscale Mechanical Characterization (PF-QNM) mode and the results are displayed in Fig. S1. The surface of the probe exhibits a flat nanotopography with an average roughness of 17.4 nm. The manufactured spearhead probes, with a thickness of 8 µm, are extremely flexible, exhibiting a Young's modulus of 2260 ± 384 MPa. This modulus is significantly lower compared with traditional silicon probes. Such reduced stiffness enhances the mechanical compatibility with brain tissue upon implantation, thereby minimizing compression and damage to surrounding brain tissues. This improvement is beneficial for mitigation of the brain FBR and enhances both the biocompatibility and the stability of long-term implants.

It is necessary to provide temporary reinforcement to ensure that the soft electrode probes are prepared for implantation. PEG reinforcement coatings of varying molecular weights (1000–14 000 g/mol) can be employed as a mechanical backbone, though they typically disintegrate within a few seconds to a few minutes [30,31] (see Table S1). Thus, PEG can be used as temporary reinforcement during rapid implantation. Alternatively, SF, as a biocompatible protein polymer, features programmable dissolution times. The degradation time of the SF coating can be controlled to be from several hours to several years [32]—a property that enables the precise placement of flexible probes during implantation surgeries. As illustrated in Fig. 1e, a mold that matches the shape of the probe was fabricated by using SU-8 and PDMS. The front side of the probe was attached to the mold and coated with a 28% wt silk protein solution and PEG (2000 g/mol) to form a 100-μm-thick temporary reinforcement layer on the back side (Fig. 1f). The SF and PEG coatings degrade at different rates, reducing both acute and chronic brain FBR [33] and enhancing the suitability of the probe for long-term implantation. Before the *in vivo* experiments, a 0.6% agarose gel model was used to simulate brain tissue, ensuring smooth and unobstructed implantation of the probe (Fig. 1g).

### Surface modification and biocompatibility characterization

**Figure 2.**
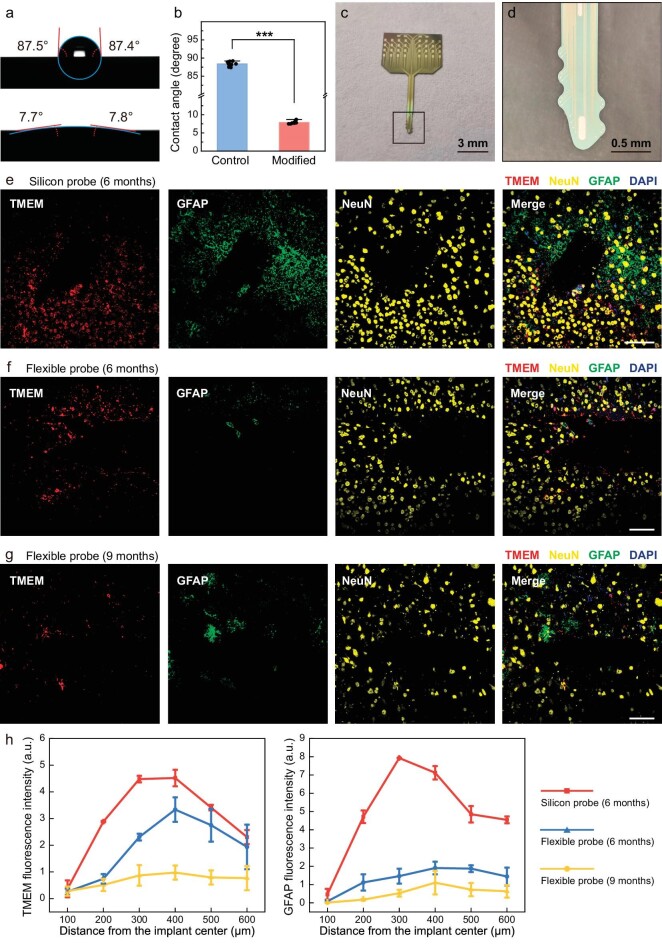
Long-term biocompatibility characterization of implantable flexible serpentine probes. (a) Surface modification of parylene film from hydrophobic to hydrophilic. (b) Alterations in the contact angle of the parylene surface pre- and post-surface modification (*n* = 8, paired *t*-test, ***P < 0.001). (c) Silicon-based electrode probes of the same shape as the flexible serpentine probes. (d) Detailed photograph of the tip of the silicon-based probe. (e) Immunohistochemical study 6 months after implantation of the silicon-based probe. Confocal fluorescence images of a 10-µm-thick brain-tissue section showing microglia (TMEM), astrocytes (GFAP), mature neurons (NeuN) and nuclei (DAPI (4',6-diamidino-2-phenylindole)). Scale bar, 100 μm. (f) and (g) Immunohistochemical studies 6 and 9 months after implantation of the flexible spearhead probes. Scale bar, 100 μm. (h) Fluorescence intensity of TMEM (left) and GFAP (right) plotted against the distance from the probe implantation center. In each group, three distinct brain slices of varying depths were taken from every animal.

When designing implantable neural electrode probes, optimization of the biocompatibility and electrical performance is crucial. This study systematically improved and tested these two key aspects to achieve higher cellular activity and enhanced signal transmission. Parylene-C membranes are highly valued in medical applications due to several advantageous properties, including low Young's modulus, high dielectric constant and minimal cytotoxicity. Additionally, the chemical inertness of parylene-C makes it an excellent material for anticorrosive applications. However, its high hydrophobicity (typically with a contact angle of >90°) and low surface energy are significant limitations [34]. Compared with hydrophobic surfaces, cells generally adhere better to hydrophilic surfaces [35]. Therefore, surface modification is essential for polymers that are used in biomaterial applications. In this study, oxygen plasma treatment (30 W, 120 s) was utilized to activate the surface of the parylene, introducing oxygen-containing functional groups such as hydroxyl and carboxyl groups. As shown in Fig. 2a and b, the hydrophilicity of the modified film surface has been significantly enhanced. The contact angle of the untreated hydrophobic surface, which was close to 90°, has been optimized to an ultrahydrophilic surface with a contact angle of ∼7.8° (refer to Supplementary Movie S1 for further details), markedly improving its biocompatibility. It has been demonstrated that neuronal cell adhesion on parylene surfaces improves by >20-fold following plasma treatment [36].

To characterize the biocompatibility of the flexible serpentine probes, we prepared silicon-based electrode probes (see Supplementary data) with identical geometries, as depicted in Fig. 2c and d, for a comparative study of long-term *in vivo* implantation outcomes. Both silicon-based and flexible probes were implanted into the brains of mice and examined by using immunohistochemical methods at 6 and 9 months post-implantation to compare lesions and immune responses. Transmembrane protein (TMEM) labeling was used to indicate the activation state of the microglia, while glial fibrillary acidic protein (GFAP) served as a marker for astrocytes, both of which are primary participants in the FBR of the brain [15], commonly used to assess neuroinflammation and glial scar formation. NeuN was used to characterize the distribution of mature neurons. As shown in Fig. 2e–g, compared with silicon-based electrode probes, the aggregation of microglia and astrocytes was significantly reduced around the flexible probes. Even after 9 months post-implantation, there was a trend for reduced inflammation around the flexible probes, suggesting that softer probes can enhance their *in vivo* applicability by reducing chronic inflammatory responses. Additionally, the areas around the probes showed a robust presence of mature neurons, indicating that neuronal integrity and numbers were well preserved. In Fig. 2h, we quantify the fluorescence intensities of TMEM and GFAP; the fluorescence intensity near the flexible probes was much lower than that near the silicon-based probes, further validating that silicon-based probes, due to their hardness and high elastic modulus, cause stronger mechanical stimulation and chronic inflammation, whereas flexible probes, with better mechanical matching, significantly reduce brain inflammatory responses. These results indicate that the flexible spearhead probes have excellent biocompatibility, are suitable for long-term *in vivo* monitoring and hold potential significance for the development of new neural interface technologies.

After hydrophilizing the surface of parylene materials, we also enhanced the detection sites with nanomaterial modifications (see Supplementary data). In this study, the electrode sites were modified with platinum nanoparticles (PtNPs) to improve electrical performance, significantly reducing impedance and phase delay (Fig. S2a and b). As depicted in the scanning electron microscope (SEM) images in Fig. S2c–e, the high specific surface area of the PtNPs provides a greater effective contact area, reducing signal attenuation and noise, and thus ensuring clearer and more reliable neural signal collection. This integrated approach of the study to the modification of both biocompatibility and electrical properties not only extends the functional lifespan of the probes, but also enhances their effectiveness for monitoring and stimulating neural activity. This is particularly important in medical and research applications in which precise and reliable neural signal recording is required.

### Long-term *in vivo* monitoring with flexible serpentine probes

**Figure 3.**
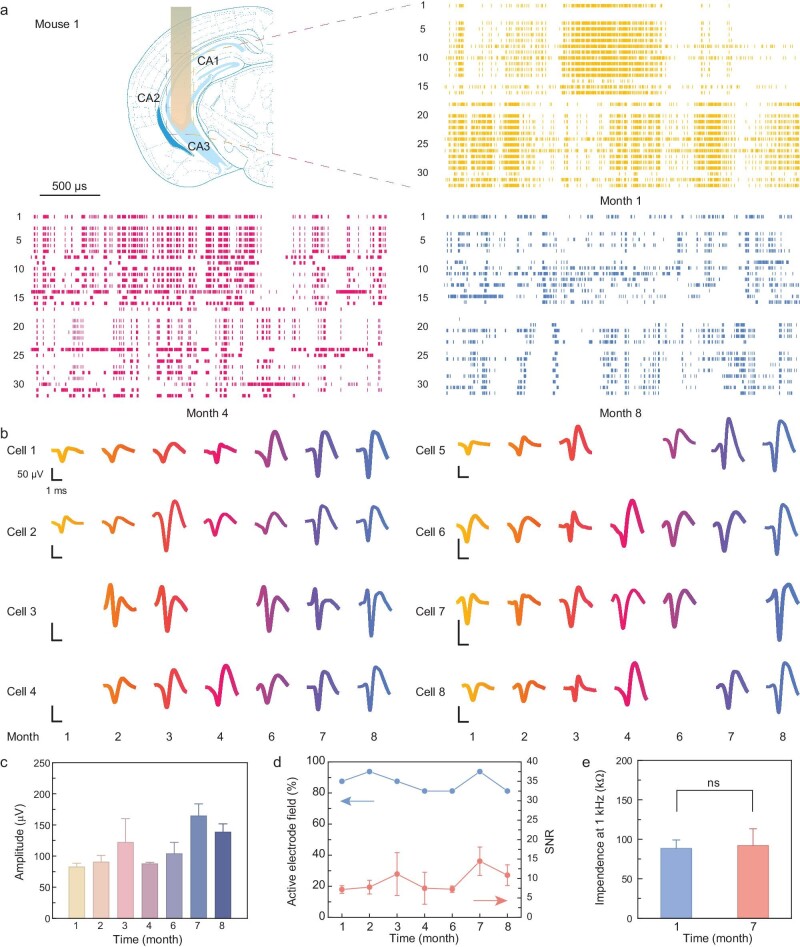
Long-term *in vivo* recording and electrical characteristics of flexible probes. (a) Electrophysiological data obtained from all electrode channels at 1, 4 and 8 months post-surgical implantation. (b) Spike waveforms recorded by the electrodes after 8 months (cell numbers are listed on the left and the time since surgery is on the bottom). (c) Changes in spike amplitude over time recorded by the electrodes. Error bars indicate SE. (d) Changes in the proportion of active electrode field (blue) and signal-to-noise ratio (red) over time. Error bars represent SE. (e) Long-term impedance changes at 1 kHz (*n* = 12, paired *t*-test). Error bars indicate SE.

After confirming the biocompatibility of the serpentine electrode probes, this study further evaluated the potential impact of long-term implantation on electrode performance. We implanted 32-channel, 8-μm-thick flexible probes into various subregions of the hippocampus in six C57/6N mice (Fig. S3). Following surgical implantation, we monitored and recorded electrical signals from the implanted probes in freely moving mice on a monthly basis. All mice demonstrated stable neural information detection for several months. Specifically, for Mouse 1, which was observed for ≤8 months, the probe was placed in the CA1, CA2 and CA3 subregions of both the ventral and dorsal hippocampus (AP = –2.7 mm, ML = –3.2 mm, DL = 3.3 mm). Most electrode channels achieved stable tracking records (Fig. 3a) and the discharge waveforms of representative neurons are shown in Fig. 3b. We used the maximum linear correlation coefficient to assess the similarity of the spike shapes at different time points, as shown in Fig. S4. This indicates the effective capture of neural activity over the study period, illustrating the reliability of the implanted probes in monitoring and recording neuronal signals. This approach allows assessment of the stability and functionality of the electrodes over time, providing insights into the durability of the implants and the consistency of signal acquisition throughout the study period. One month post-implantation, a single probe recorded action potentials from 50 neurons in the hippocampal subregions CA1, CA2 and CA3. Eight months later, we revisited various hippocampal subregions and collected discharge data from 47 neurons. Some channels exhibited increased correlation in the detected field potentials (Fig. S5), which may suggest enhanced synchronization of neural activity or changes in functional connectivity during long-term recording. The power spectral density frequency distribution trends of the LFP at different months were generally consistent (Fig. S6), with similar attenuation at higher frequency bands and overall spectral shapes. This indicates that the electrode captures similar neural network activity patterns over extended periods. Furthermore, we implanted flexible probes into additional locations within the mouse hippocampus and similarly obtained months of long-term, stable recordings (Fig. S7).

Over the course of recording, the flexible serpentine electrode probe captured high-quality neuronal action potentials and we noted a gradual increase in their amplitude (Fig. 3c). Importantly, both the active channel ratio and the signal-to-noise ratio (SNR) of the flexible serpentine probe remained satisfactory throughout the extended 8-month period (Fig. 3d). Specifically, the proportion of active channels decreased slightly from 87.5% in the first month to 81.25% by the eighth month, while the SNR consistently remained at >7. This performance demonstrates the durability and reliability of the flexible probes in capturing clear neural signals over prolonged periods, underscoring their potential for long-term neurological studies and applications. Moreover, the stability of the electrical properties of the probes was also assessed (Fig. 3e). The impedance of the electrode sites, modified with PtNPs, showed no significant changes at an electrophysiological frequency of 1 kHz. These findings underscore that our flexible serpentine probes are capable of stable, long-term neural information recording across multiple brain regions, making them highly valuable for longitudinal studies of significant neurological disorders such as epilepsy.

### Behavioral validation of cognitive impairment due to TLE

**Figure 4.**
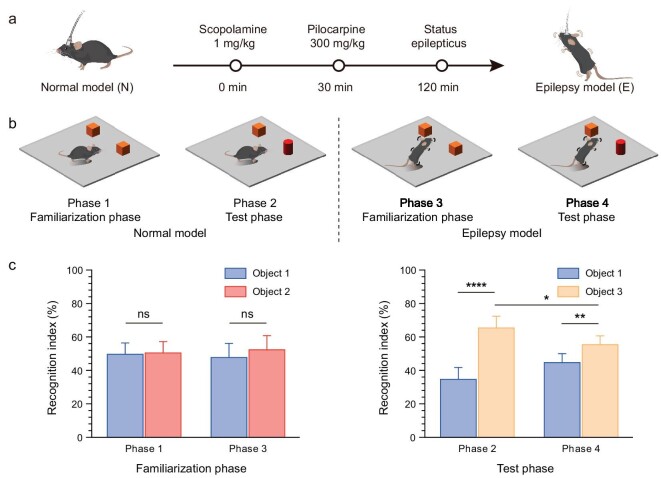
TLE mice exhibit impaired cognitive abilities in the NOR tests. (a) Epilepsy modeling process. (b) NOR test flow showing familiarization and testing phases for both normal mice (left) and epileptic mice (right). (c) Recognition index for the two objects in the familiarization (left) and testing phases (right) for normal and epileptic mice (*n* = 6, paired *t*-test, *P < 0.05; **P < 0.01; ****P < 0.0001). Error bars indicate SE.

Epileptic seizures are the most prominent symptom of epilepsy, yet both focal and generalized types of epilepsy are associated with similar complications, such as cognitive impairments. Cognitive impairment in epileptic animals was confirmed through behavioral experiments. Initially, TLE was modeled as outlined in Fig. 4a by using a combination of scopolamine and pilocarpine to induce sustained epileptic seizures, resulting in a chronic TLE mouse model (refer to Supplementary methods and Movie S2). To assess cognitive deficits, we utilized a novel object recognition (NOR) test to evaluate changes in the spatial cognitive abilities of mice before and after the induction of TLE (Fig. 4b). The NOR test is particularly valuable in the context of TLE research, as the temporal lobe, especially the hippocampus, plays a crucial role in the formation of spatial memory. The study was designed to include four sessions of the NOR tests that comprised the familiarization and test phases for both normal and post-epilepsy modeling in mice. The movement trajectories of mice during NOR trials are shown in Fig. S8. In the familiarization phase (Fig. 4c, left), no significant differences were observed in the recognition index (preference index) between normal and epileptic mice for identical-looking objects (Objects 1 and 2). During the testing phase (Fig. 4c, right), both normal and epileptic mice exhibited a preference for the new Object 3, indicating that memories of familiar objects were retained in the epileptic animals. Nevertheless, a significant decline in the recognition index for the new object was observed in the NOR test in mice with TLE compared with their normal state. This serves as direct evidence of spatial memory impairment caused by temporal lobe epilepsy, confirming that TLE indeed impairs cognitive abilities in animals.

### Epilepsy disrupts spatial encoding by place cells

**Figure 5.**
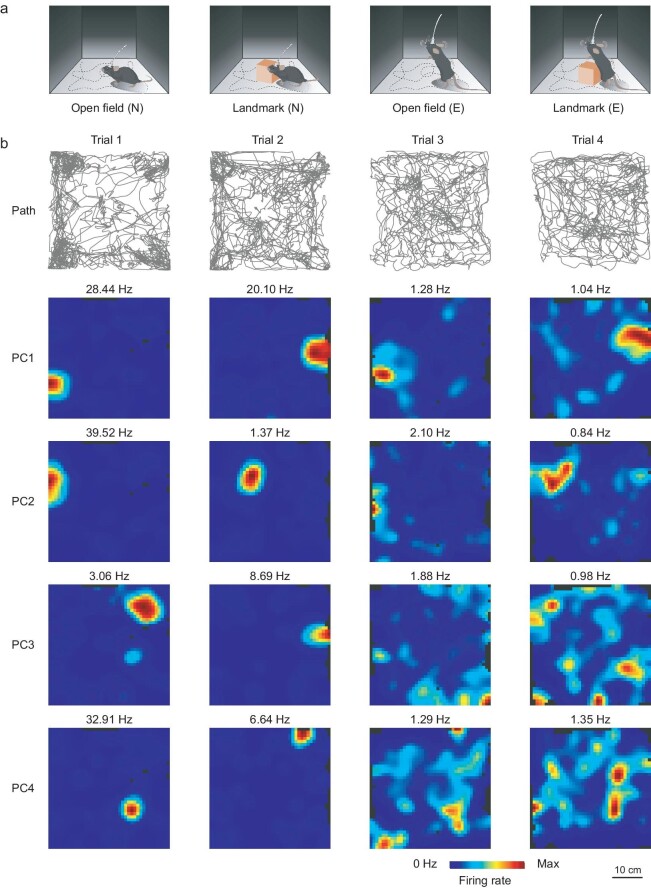
Abnormal anchoring and remapping of spatial place fields in epileptic mice. (a) Schematic diagram of the open-field exploration task. Normal mice (left two images) and epileptic mice (right two images) undergo exploration in an open field with and without landmarks. (b) Representative spatial firing rate heat maps. The traces represent the movement trajectories of the animals during the four trials (top row). The heat maps below display the firing activity from the place cells of the mice. Each row represents one place cell (place-cell ID labeled on the left). The color scale is adjusted to the maximum peak rate for each of the four trials, with the peak rate for each trial labeled above. The color scale is in the lower right corner.

Epilepsy is closely associated with gene expression, apoptosis, brain damage and other factors. These physiological and molecular changes not only trigger seizures, but also lead to cognitive dysfunction [37]. Therefore, this study investigates changes before and after the onset of epilepsy at the single-cell level, with a particular focus on the place cells that are responsible for spatial representation in the hippocampal region, which is affected by TLE. We implanted the flexible serpentine probe into the CA1 region of the mouse hippocampus and allowed the mice to move freely in open fields with and without landmarks (Fig. 5a) to observe changes in place-cell-firing patterns before and after epilepsy induction.

During open-field exploration activities in normal mice, we recorded neuronal discharges from 32 neurons. After screening, 15 of these neurons met the criteria for place cells (see Supplementary data for place-cell determination criteria). When mice were active in an open field with landmarks, we recorded discharges from 19 place cells. However, during the activity of TLE animals, only eight of the previously recorded place cells were tracked. Among these, seven place cells (46.7%) were not detected, which could be because these cells became inactive after the induction of epilepsy or were not activated during the spatial task. The discharge patterns of these eight continuously tracked place cells, labeled PC1 to PC8, are displayed in Fig. 5b and Fig. S9. During the first trial of the spatial exploration task in the open field, place cells fired only at specific locations within the environment. For individual place cells, global remapping is characterized by significant changes in both firing rates and firing locations. Global remapping represents the ability of normal place cells to encode new environments, reflecting their normal functioning to some extent. To demonstrate this, we introduced an orange cube as a landmark in Trial 2. When environmental cues were altered, place-cell discharges exhibited clear global remapping, indicating their adaptive response to changes in the spatial context. When we repeated the spatial exploration tasks (Trial 3 and Trial 4) 3 days after modeling epilepsy in mice, we observed a stark contrast in the performance of place cells compared with previously formed patterns. While some place cells, such as PC1 and PC2, retained their place fields anchored at the same locations after the epilepsy induction, maintaining the capability for global remapping, others displayed a deviation from their initial state. Place cells such as PC3 and PC4 generated place fields at multiple locations across the space, indicating a disruption in their spatial-coding capabilities.

**Figure 6.**
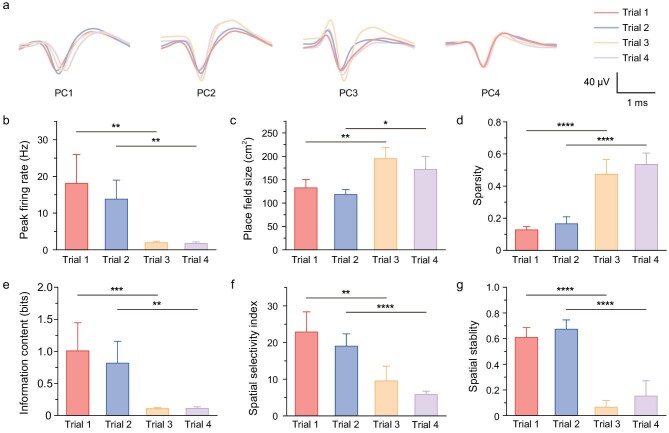
Impact of epilepsy on spatial encoding by place cells. (a) Changes in PC1–PC4 spike firing waveforms across four trials. (b) Changes in peak firing rates of place cells across four trials. (c) Changes in the area of place-cell spatial fields across four trials. (d) Changes in the sparsity of place-cell firing across four trials. (e) Changes in the information density of place cells across four trials. (f) Changes in the spatial selectivity index of place cells across four trials. (g) Changes in the spatial stability of place cells across four trials (*n* = 8, paired *t*-test, *P < 0.05; **P < 0.01; ***P < 0.001; ****P < 0.0001). Error bars represent SE.

To assess changes in place-cell spatial encoding before and after epilepsy, we quantified several typical parameters (see Supplementary data for detailed calculation methods) and present the results in Fig. 6. Across the four trials, place-cell-firing waveforms remained consistent (Fig. 6a and Fig. S9), verifying that the flexible serpentine electrode probes could be anchored in brain tissue for stable tracking of neuronal firing. We first calculated the peak spatial firing rate (Fig. 6b), finding a significant reduction in epileptic mouse place-cell-firing rates compared with normal states. To quantify the trend for the firing fields to become more dispersed and increase in size, we calculated the area of the firing fields (Fig. 6c) and the sparsity index (Fig. 6d). Sparsity represents the distribution characteristics of place-cell-firing activity in the exploration area. The results showed a significant increase in the place field area and sparsity index after epilepsy. To assess the mean information content of neurons and the specificity of the responses of the place cells to specific areas in spatial encoding, we measured the spatial information content (Fig. 6e) and spatial selectivity index (Fig. 6f)—both of which significantly decreased compared with pre-epileptic states. Finally, we evaluated the reproducibility of spatial firing patterns and Fig. 6g shows the stability of the place cells. Spatial firing stability is crucial for spatial encoding and the significant decrease in stability further confirms the disruption of place-cell function by epilepsy.

Place-cell activity and their normal spatial encoding are crucial for spatial navigation and cognition. The impairment of spatial encoding in epileptic mice helps to explain their poor performance in the NOR test. In summary, our experiments demonstrate that spatial selectivity, stability and neuronal information content are all impaired in mice with epilepsy. The results confirm that continuous epileptic seizures cause significant disruption to the functionality of place cells. This underscores the profound impact of epilepsy on the cognitive capabilities that are related to spatial memory and navigation, highlighting the need for further studies to explore mechanisms and potential interventions to preserve or restore neuronal function in the context of epilepsy.

## DISCUSSION AND CONCLUSION

In this research, we developed and fabricated a flexible serpentine electrode probe by using parylene-C as the substrate, with the aim of monitoring neuronal discharges in the hippocampal region of mice. The wavy shape, resembling a curved snake body, facilitates stable anchoring of the flexible electrode probe in brain tissue. The ultra-thin and soft characteristics of the probe match the mechanical properties of brain tissue, substantially reducing potential brain damage and brain FBR compared with more rigid implants and enhancing the capability for prolonged motion detection. Surface modification of the parylene film to create an ultrahydrophilic surface significantly enhances the adhesion and biocompatibility of neuronal cells on the surface. Additionally, this study utilized SF-PEG dorsal coating as temporary reinforcement during the implantation of the probes. The rapid metabolism of PEG aligns with an early biological response strategy, reducing acute reactions associated with mechanical trauma at the implantation site [33], while enabling continued cellular growth and proliferation [38]. The untreated hydrophobic backside of the probe facilitates the attachment and prolonged retention of SF—a biocompatible material. The SF coating degrades over a period of days to weeks, thereby mitigating the chronic inflammatory response that is triggered by the long-term presence of foreign materials [39].

The integration of these properties into a single flexible electrode probe significantly reduces the severity and duration of post-implantation brain FBR [15]. Due to the ease of integrating bioactive factors into SF and PEG hydrogels, optimization of existing strategies by the incorporation of dexamethasone [40], the neural adhesion molecule L1 [41] and extracellular matrix [42] components could further reduce glial activation and immune inflammation. This approach may further reduce glial activation and immunoinflammatory responses, potentially enhancing outcomes. To date, we have successfully conducted ≤8 months of *in vivo* electrophysiological recordings in mice, capturing high-quality neuronal discharges. The flexible serpentine electrode probe demonstrated excellent stability, showing no significant inflammatory responses even after long-term implantation and exhibiting outstanding biocompatibility. In terms of electrical performance, the flexible probes maintained a stable proportion of active channels and SNR, with no significant changes in impedance at electrophysiological frequencies (1 kHz). In conclusion, our study underscores the potential of surface-modified flexible serpentine probes to provide stable recordings across multiple brain regions and over extended periods. This holds significant implications for the advancement of long-term brain disease detection studies and the development of brain–computer interfaces.

This study utilized the flexible device that was implanted into the hippocampus of epileptic mice to explore the neural mechanisms that underlie cognitive impairment associated with epilepsy. It is widely recognized that epileptic seizures and cognitive impairments are causally linked, and the neurological changes that are induced by epilepsy are primary contributors to cognitive decline. To assess cognitive function, we conducted a series of NOR tests that leverage the inherent preference of mice for novel objects without the need for external rewards or punishments. The results indicated that, although epileptic mice maintained a natural curiosity towards new objects, their recognition indices were significantly lower compared with those in their pre-epileptic state, illustrating a marked impairment in cognitive abilities due to temporal lobe seizures.

Research in both rodents and primates has shown that the process of object familiarity assessment hinges on the integrity of the medial temporal lobe—particularly the hippocampus [43]. During the NOR task, the reconsolidation phase—in which memories such as spatial or contextual characteristics of an object are recalled in the presence of a novel item—depends critically on hippocampal function [44]. It is established that the firing activity of hippocampal place cells and the rate of neurogenesis [45] are integral to the formation and consolidation of spatial memories. Our study sought to delineate the cellular mechanisms that contribute to spatial cognitive impairments by examining the spatial encoding capacity of place cells before and after the onset of epilepsy. In spatial exploration tasks, nearly half of the place cells were either inactivated or failed to activate post-epilepsy. Among the units that were continuously tracked, most place cells exhibited a deviation in their firing fields compared with pre-epilepsy, which may be attributed to the frequent significant changes in the place fields of epileptic mice over time [8]. Notably, even in place cells for which the place field remained anchored and global remapping was preserved, discharges were scattered. In epileptic animals, both the area and the sparsity of the place fields were significantly increased. Furthermore, the spatial information content and spatial selectivity substantially declined, consistently with prior research [46]. The observed decline in performance may be attributed to the substantial loss of interneurons within days following treatment with pilocarpine [47], leading to deficits in spatial memory, which relies on the precise timing and excitatory information processing of these interneurons. This disruption in the ability of the circuit to properly balance excitation and inhibition could explain the observed reduction in peak spatial firing rates. Additionally, the silencing of place cells may cause spatial regions that should be encoded to be overlooked. The aberrant discharges that are induced by epilepsy disrupt the information processing of place cells, altering ongoing cognitive processes and resulting in transient cognitive impairments. Some clinicians believe that the repetitive transient damage that is caused by interictal discharges has a pervasive impact on cognitive functions [48]. Our findings corroborate this theory, demonstrating a significant reduction in the stability of place-cell discharges in epileptic animals, supporting the pervasive impact of epilepsy on cognitive capabilities.

The occurrence of epileptic seizures alters the information-processing mechanisms of hippocampal place cells, impairing spatial-cell encoding. When a substantial number of neurons that are associated with spatial cognition are affected, this effect can have magnified consequences [37], leading to abnormalities in spatial encoding and resulting in spatial cognitive impairments. Future long-term studies of epilepsy will further elucidate the system-level changes that result from altered cellular firing activity. Additionally, examination of the timeline of spatial-coding deficits may provide insights into cognitive impairment circuits, potentially helping to mitigate cognitive deficits in patients with epilepsy.

However, there are some limitations in this study that need to be considered. First, although our flexible snake-shaped electrode probe demonstrated good stability and biocompatibility in mice, its long-term *in vivo* stability requires further verification. Potential degradation of the materials may affect the electrical performance and recording quality of the probe, especially over longer timescales. Currently, research on the *in vivo* degradation time of SF at different concentrations is still scarce. It would be meaningful in the future to systematically study the effects of different combinations of SF and PEG on the performance of the reinforcement layer, as well as their *in vivo* degradation and tissue responses. Additionally, our study primarily focused on mouse models. Future research should aim to evaluate the performance of the probe over longer implantation periods and in different animal models, exploring methods of material modification and structural optimization to enhance the long-term stability and functional reliability of the probe.

## METHODS

### Preparation process of flexible electrode probes

The fabrication process of the flexible serpentine electrode probe (Fig. 1d) is as follows. (i) Parylene-C base preparation: using a standard 4-inch silicon wafer in a vacuum deposition system, a 5-μm-thick transparent parylene-C film is deposited as a base. (ii) Patterning conductive layer: a 1.2-μm-thick layer of AZ 5214 negative photoresist is spin coated onto the parylene-C film. The pattern of the conductive layer is then defined by exposing and developing the photoresist through a mask. (iii) Metal-layer deposition: a Cr/Au (30 nm/200 nm) metal layer is deposited onto the patterned surface by using an electron beam evaporation process. (iv) Lift-off process: a lift-off process is performed to remove excess metal, forming the conductive lines. (v) Insulating layer: chemical vapor deposition is used to coat a 3-μm-thick parylene-C insulating film over the conductive layer. (vi) Exposing electrode sites and pads: a 6-μm-thick layer of positive photoresist AZ 4620 is spin coated. The photoresist is exposed and developed through an insulating layer mask to define the positions of the electrode sites and pads. Oxygen plasma etching is used to remove the parylene-C insulation layer, thereby exposing the sites and pads. (vii) AZ 4620 serves as a mask to shield the non-exposed areas from oxygen plasma etching. (viii) ICP-CVD (Inductively Coupled Plasma Chemical Vapor Deposition) etching: the flexible shape of the probe is etched by using ICP-CVD. To protect the base, a 12.5-μm-thick layer of AZ 4903 is applied. (ix) Residue removal: after etching, any residual photoresist is removed, resulting in independent, flexible probes with smooth edges and no signs of burning. The probe shank dimensions are 5.6 mm × 512 μm × 8 μm (length × width × thickness) and the pad area measures 4 mm × 5.65 mm × 8 μm (length × width × thickness).

### PDMS mold fabrication

SU-8 coating: on a 4-inch standard silicon wafer, a 100-μm-thick layer of SU-8 2075 is spin coated. The resist is developed and baked to obtain a pattern that is slightly wider than the probe. PDMS casting: the silicone elastomer base and curing agent are mixed at a 10:1 weight ratio. The mixture is allowed to stand for 2 hours to remove bubbles, then the PDMS liquid is poured onto the SU-8 pattern and heated 80°C for 3 hours to cure and obtain a 100-μm-deep PDMS mold.

### Subject

This study used 14 female C57BL/6N mice which were aged >8 weeks (6 for long-term *in vivo* testing, 6 for epilepsy modeling and NOR tests, and 2 for epilepsy modeling and spatial exploration). When the mice reached a weight of >20 grams, a flexible electrode probe was implanted into the hippocampal region via craniotomy. After a recovery period of >1 week, electrophysiological data collection and behavioral tests were conducted. The mice were housed separately in a controlled environment with alternating periods of light and darkness, and maintained at a temperature of ∼22°C and 40–50% humidity. All training and experiments were carried out during their dark period. The animal ethics guidelines were strictly followed during the procedures and they received approval from both the Beijing Experimental Animal Protection Association and the Committee for the Care and Utilization of Animals at the Aerospace Information Research Institute.

## Supplementary Material

nwae402_Supplemental_Files
