# Supplementary material for: Chronic implantable flexible serpentine probe reveals impaired spatial coding of place cells in epilepsy
Source: Natl Sci Rev. 2024 Nov 15;12(2):nwae402. doi: 10.1093/nsr/nwae402 (PMC11737403; doi:10.1093/nsr/nwae402)
Supplement: nwae402_Supplemental_Files [file nwae402_supplemental_files.zip › Supplementary data.docx]

Supplementary data for

**Chronic Implantable Flexible Serpentine Probe Reveals Impaired Spatial Coding of Place Cells in Epilepsy**

Yu Wang1,2, Meiqi Han1,2, Zhaojie Xu1,2, Shiya Lv1,2, Gucheng Yang1,2, Fan Mo1,2, Luyi Jing1,2, Qianli Jia1,2, Yiming Duan1,2, Wei Xu1,2, Peiyao Jiao1,2, Yaoyao Liu1,2, Jin Shan1,2, Ming Li1,2, Mixia Wang1,2, Jinping Luo1,2, Yilin Song1,2, Juntao Liu1,2,*, Yirong Wu1,2,*, Xinxia Cai1,2,*

***Corresponding author:** Xinxia Cai (xxcai@mail.ie.ac.cn); Yirong Wu (wyr@mail.ie.ac.cn); Juntao Liu (liujuntao@mail.ie.ac.cn).

Supplementary Methods

**Packaging process of flexible electrode probes**

The flexible serpentine probe packaging process is as follows. Using a low-temperature solder ball welding process, design the solder joints on the flexible printed circuit (FPC) to match the electrode pad arrays. Place 200-micron-diameter low-temperature solder balls on the FPC pads and melt them on a heating stage set at 200°C. Align the electrode pads with the FPC solder balls, and heat at 220°C until firmly welded. Finally, apply silicone rubber to protect the pad area, completing the probe packaging.

**Preparation process of silicon-based electrode probes**

The fabrication process has been previously reported in detail [1,2]. In short, a 500 nm silicon dioxide insulation layer is prepared on a 4-inch standard silicon wafer via thermal oxidation. Following photolithography to form conductive layer patterns, the lift-off process involves sputtering and stripping Ti/Pt (30 nm/250 nm) to serve as the conductive metal layers. Then, deposit a 300 nm silicon dioxide layer and a 500 nm silicon nitride layer sequentially over the conductive layer via chemical vapor deposition. Define the electrode site and pad patterns through photolithography and expose the windows by plasma etching, then deep-etch to form the probe shape. Lastly, obtain the silicon-based electrode probes through a self-stopping wet etch process.

**Measurement of micromechanical properties**

Fix the flexible probe onto glass slides, and use a Bruker Dimension Icon AFM instrument to scan a 2 μm × 2 μm area of the sample. Measurements are performed using the PF-QNM mode. This mode provides a quantitative analysis of the mechanical properties at the nanoscale for the flexible probes, gathering information on parameters such as probe height, peak force error, DMT modulus, adhesion, indentation, and dissipation.

**Modification of MEA**

The modification of PtNPs was performed according to the following procedures. In brief, the plating solution was prepared by mixing 48 mM H2PtCl6 and 4.2 mM Pb(CH3COO)2 at the 1:1 ratio. In the 10 ml plating solution, the working electrode of MEA and the auxiliary electrode of platinum filament formed a two-electrode configuration. PtNPs was electro-deposited via chronoamperometry on an electrochemical workstation. The standard voltage was 1.2 V and the time of duration was 45 s.

**Fabrication of silk fibroin films**

Silk fibroin is a biopolymer that serves as a temporary soluble support material, forming a strong and flexible transparent film [3]. It is biologically absorbable and exhibits programmable dissolution rates. The silk fibroin used in this work is extracted from silkworm cocoons following a published procedure [4]. Silk fibroin induces a mild immune response. To eliminate the fibroin, silk cocoons undergo a process of chopping and boiling in a sodium carbonate solution (0.02 mol/L) for a duration of 40 minutes. The silk is then repeatedly rinsed three times with cold deionized water, excess water is squeezed out, and the silk is spread on aluminum foil to dry overnight in a fume hood. The extracted sericin is dissolved in a LiBr solution (9.3 mol/L) and dissolved at 60°C for 4 hours to yield a 20 wt% high-viscosity, amber-colored, transparent lithium bromide silk solution. Transfer the solution into a 3.5 kDa dialysis membrane for dialysis over three days, changing the water six times to remove LiBr. Centrifuge the solution at 12000 rpm for 15 minutes to remove the white precipitate, repeating once. Finally, concentrate the solution in a vacuum desiccator to obtain a 28 wt% solution. Pour this solution onto the probe's back side and air-dry for 10 hours to form an even film of several tens of microns thick.

**Electrical Performance Characterization of Electrodes**

An electrochemical workstation was used in a three-electrode system, where the electrode sites served as the working electrode, a platinum wire acted as the counter electrode, and an Ag/AgCl electrode was used as the reference electrode. All electrodes were immersed in PBS solution to perform impedance spectroscopy characterization. Impedance and phase curves of the electrode sites were obtained before and after modification over a frequency range from 10 Hz to 106 Hz.

**Surgical Procedure**

Mice were anesthetized with isoflurane to ensure they were pain-free during the surgery. They were secured on the surgical table using ear bars to stabilize the head. After removing the scalp to expose the skull, a craniotomy was performed using a skull drill to remove a portion of the bone, exposing the brain tissue above the target area, as shown in **Fig. S3**. Subsequently, four skull screws were fixed onto the skull surface, and copper wires were used to connect the electrode ground. Based on the brain atlas, the electrode was implanted into the target brain region using a micropositioner (KOPF Model 2662 Direct Drive Micropositioner). Finally, the skull was sealed with dental cement, and appropriate postoperative care was provided.

**TLE model induction**

The pilocarpine model is a chronic TLE model that closely mimics human disease [5]. The preparation process is shown in **Fig. 4a.** Initially, mice received intraperitoneal injections of methyl-scopolamine (1 mg/kg, i.p.) to block the peripheral cholinergic effects of pilocarpine, while not interfering with the development of SE and chronic epileptic seizures. Thirty minutes later, inject pilocarpine (300 mg/kg, i.p.) to induce SE (movie S2). After one hour of SE, the mouse TLE model is considered successfully established.

**Novel object recognition (NOR) test**

The NOR test measures cognitive memory abilities in mice by comparing the exploration time between familiar and novel objects. Recognizing novel objects requires greater cognitive skills than exploring new environments or individual objects [6]. After the mice acclimatize to the environment, the NOR test is conducted in two phases: the familiarization phase and the test phase (**Fig. 4b** and **Fig. S7a**). Familiarization Phase (Phase 1): Within a black acrylic open-top cubic box (side length 40 cm), two identical orange cubes (side length 3 cm), designated as Object 1 and Object 2, were placed and fixed at a distance of 10 cm from the side walls. Introduce the mouse into the box, back facing the objects, equidistant from both, and allow 5 minutes for free exploration. Test Phase (Phase 2): Replace Object 2 with a completely different red cylinder (3 cm in diameter and 6 cm in height) and let the mouse freely explore for another 5 minutes. After TLE is induced in the mice, the NOR test is repeated following a 3-day recovery period (as shown in Phase 3 and 4). There is a 10-minute interval between the familiarization and test phases. After each NOR session, clean the box and objects with a 75% ethanol solution to prevent olfactory cues from affecting behavior. It should be noted that the experimental environment required uniform illumination, controlled temperature and humidity, and low noise levels to avoid interference from external factors.

A camera system and software are used to record the exploration time (ET) of each mouse on each object to assess cognitive ability. Exploration is defined as the mouse pointing its nose within 3 cm of the object, sniffing, or touching it. To minimize observer bias, behavioral recordings were conducted by experimenters who were blinded to the experimental groupings. The recognition index (RI) of object 1 is calculated using the following formula.

**Open field exploration test**

The experimental field is an open-top black acrylic box (40 × 40 × 40 cm). The box is shielded with a copper mesh connected to the ground to reduce noise interference. Mice explore the field for 1 hour daily for a week to familiarize themselves with the environment. The experimental procedure is illustrated in **Fig. 5a**. Initially, mice equipped with signal transmission cables were allowed to freely explore an open field for 10 minutes (**Trial 1**). Subsequently, an orange cube (3 cm in length) was placed at the center of the open field as a landmark to alter environmental cues, thereby assessing the remapping function of place cells following changes in environmental information. After being returned to their cages for a 20-minute rest, the mice were reintroduced to the experimental arena to explore for another 10 minutes (**Trial 2)**. Following TLE modeling, the mice recover for two days before repeating the aforementioned exploration sessions (Trial 3 and Trial 4). At the start of each trial, the mouse was placed at the same location on the edge of the open field. Neural information from the hippocampal CA1 region of both normal and epileptic mice is monitored using flexible electrode probes, while a camera system records the mice’s activity. Electrophysiological data from the probes are captured using a neural information detection system (AIRCAS-128, China) at a sampling rate of 30 kHz, and mouse movement trajectories are extracted using behavioral analysis software (EthoVision XT 16, Noldus, China). During the experiments, biscuit crumbs are placed in the open field and the cables connected to the mice’s heads are slightly agitated to encourage exploratory behavior.

**Immunohistochemistry and imaging**

Mice were anesthetized with isoflurane and perfused intracardially with physiological saline. The complete brains (**Fig. S10**) were extracted and submerged in 4% PFA solution and refrigerated overnight. The fixed brain tissue was then embedded in paraffin and sectioned transversely to water. Sections were microwaved in EDTA (pH = 8.0) buffer for antigen retrieval. 3% H2O2 was applied to the sections to cover the tissue and incubated at 25 ℃ for 25 minutes to block endogenous peroxidases, reducing non-specific background staining. This was followed by application of 3% BSA to block for 30 minutes. Rabbit anti-GFAP antibody (16825-1-AP, Proteintech, 1:100 dilution) was applied to cover the tissue and incubated at 4 ℃ in a moist chamber. Then sections were incubated for 50 minutes in Goat Anti-Rabbit IgG (1:500 dilution). 50-100 µL of TSA-488 staining solution was applied and incubated for 10 minutes. The sections were again microwaved in EDTA to remove the bound primary and secondary antibodies, then incubated with rabbit anti-NeuN antibody (ab177487, Abcam, 1:100 dilution). Then the sections were incubated for 50 minutes in Goat Anti-Rabbit IgG (1:500 dilution). 50-100 µL of TSA-555 staining solution was applied, incubated for 10 minutes, and microwaved. Sections were then incubated with rabbit anti-TMEM119 (ab209064, Abcam, 1 µg/ml), followed by incubation for 50 minutes in Goat Anti-Rabbit IgG (1:500 dilution). An amount of 50-100 µL of TSA-647 staining solution was administered and allowed to incubate for a duration of 10 minutes. Subsequently, DAPI staining solution was administered, and the sections were subjected to an incubation period lasting 10 minutes. Finally, an anti-fading mounting medium was applied, and images were captured using a laser scanning confocal microscope (LSM700, ZEISS).

**Calculation of Place Fields**

To analyze the discharge of place cells using neural information obtained from spatial exploration tasks, the following steps are taken: First, data preprocessing involves filtering neuronal discharges occurring during time intervals with instantaneous movement speeds ranging from 2.5 cm/s to 100 cm/s [7]. Next, place fields are identified by smoothing the position data and dividing it into 1 cm × 1 cm bins. The total number of spikes in each spatial grid is calculated, divided by the time the animal spends in that bin, and Gaussian smoothing is applied to obtain the spatial discharge rate per bin. An adaptive smoothing method is employed to balance between blurring errors and sampling errors. Rate heatmaps comprising 40 × 40 spatial bins are constructed based on the pulse rates in each grid. The peak firing rate is defined as the maximum value of the spatial pulse distribution. A place field is defined as a continuous area of at least 80 square centimeters where the pulse rate exceeds 200% of the average spatial discharge rate. The size of the place field is determined by multiplying the size of each spatial bin (1 square centimeter) by the total number of grids covered by the cell's place field. This methodology ensures robust identification and characterization of place fields while considering factors such as spatial resolution and variability in neuronal activity.

**Sparsity**

The sparsity index was defined as:

Here, represents the spatial discharge rate of the *i*-th bin, denotes the probability of the animal appearing in the current bin (determined by dividing the time of activity in the *i*-th bin by the total recording time), and represents the average spatial discharge rate.

**Information content**

We assessed the contribution of each neuron to the overall scene in the spatial exploration task by computing the information rate at each location, where information content represents the amount of information carried by each neuron. The Information content [8] of the spatial neural activity rate map (in bits) of a single neuron was defined as:

Here, represents the spatial discharge rate of the *i*-th bin, denotes the probability of the animal appearing in the current bin, and represents the average spatial discharge rate.

**Spatial selectivity index**

The spatial selectivity index [9] is defined as:

**Spatial stability**

Spatial stability was estimated by comparing the firing patterns between the first and second halves of each trial. During a 10-minute single-cell recording session, each trial was divided into two 5-minute halves. The rate maps for each half were smoothed and binned into a matrix of 1 cm × 1 cm bins. Spatial stability was defined as the correlation between spatial discharge rates in the bins of the two halves of the trial [7].

**Place cell criteria**

Based on shuffled distributions, neurons were classified as place cells only if their information content and stability of discharge were both significantly higher than chance (P < 0.05), and they exhibited continuous place fields.

Supplementary Figures


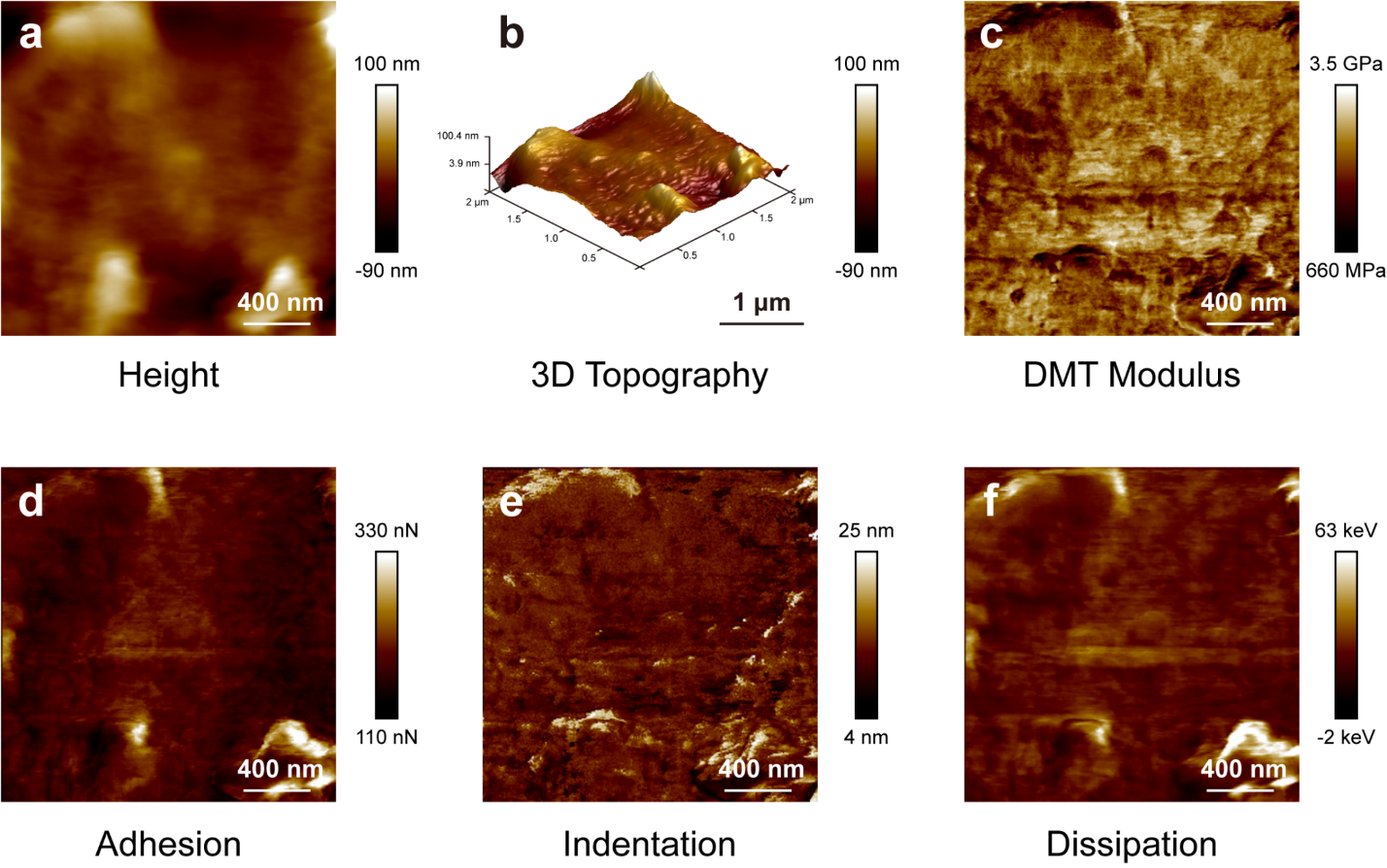


**Figure S1. PF-QNM mode characterization of imaging information in a 2 μm × 2 μm area.** Using AFM, data on height, peak force error, DMT modulus, logarithmic DMT modulus, adhesion, indentation, and dissipation were collected. **(a, b)** Two-dimensional and three-dimensional surface morphologies of the sample. The average surface roughness is 17.4 nm, indicating a uniform surface. **(c)** The probe is extremely soft, with a DMT modulus of 2260 ± 384 MPa. The DMT modulus, an estimate of Young's modulus, is obtained by fitting the force curve using the DMT model. **(d)** Adhesive force of the material surface. **(e, f)** Material indentation and energy dissipation.


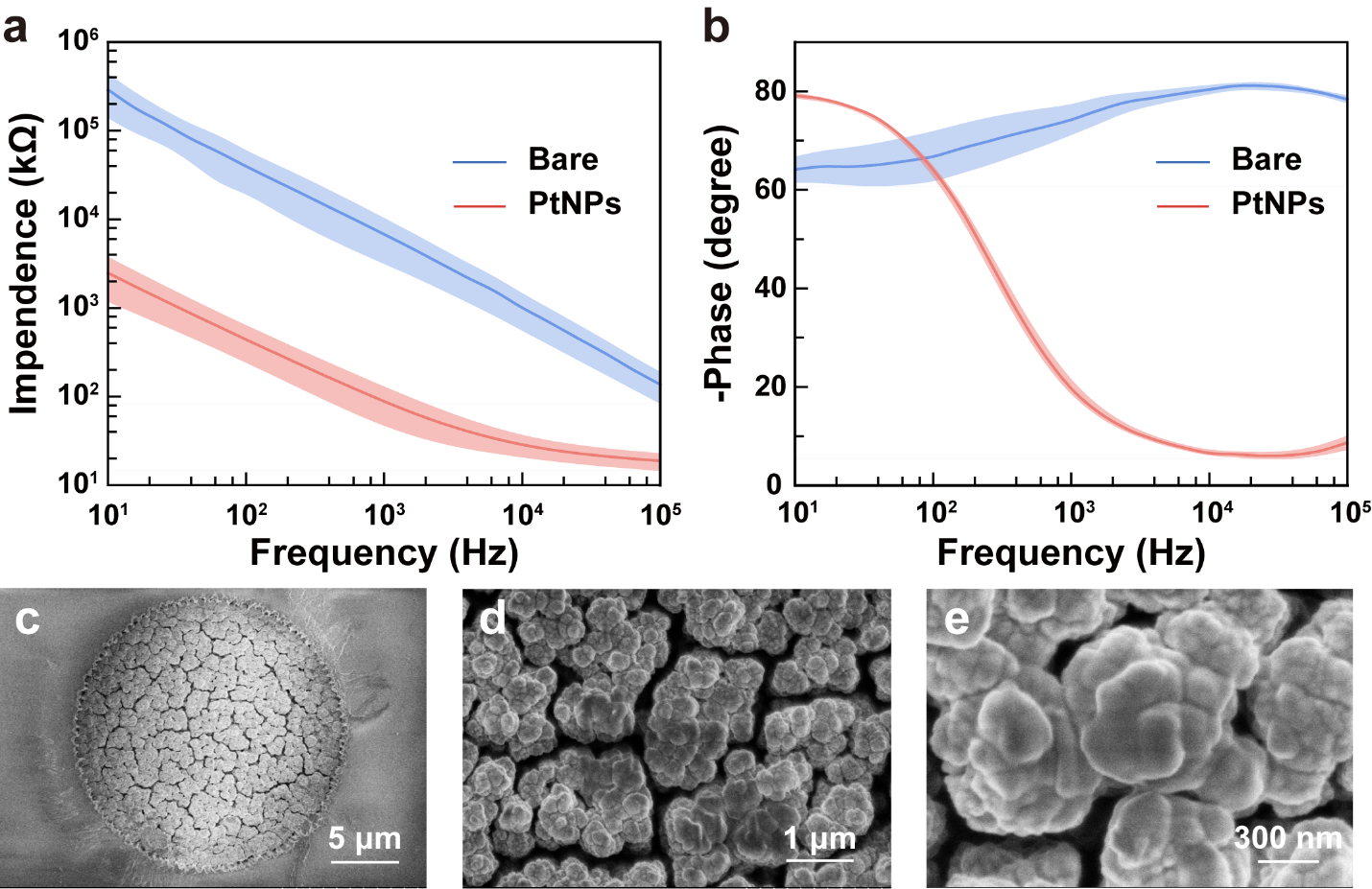


**Figure S2. Electrical characteristics of the flexible electrode probes. (a, b)** Impedance and phase of the bare surface compared to the surface modified with platinum black nanoparticles. **(c)** SEM image of the surface after modification with PtNPs. **(d, e)** Five-fold and fifteen-fold magnifications of the image in **c**, respectively, showing detailed views.


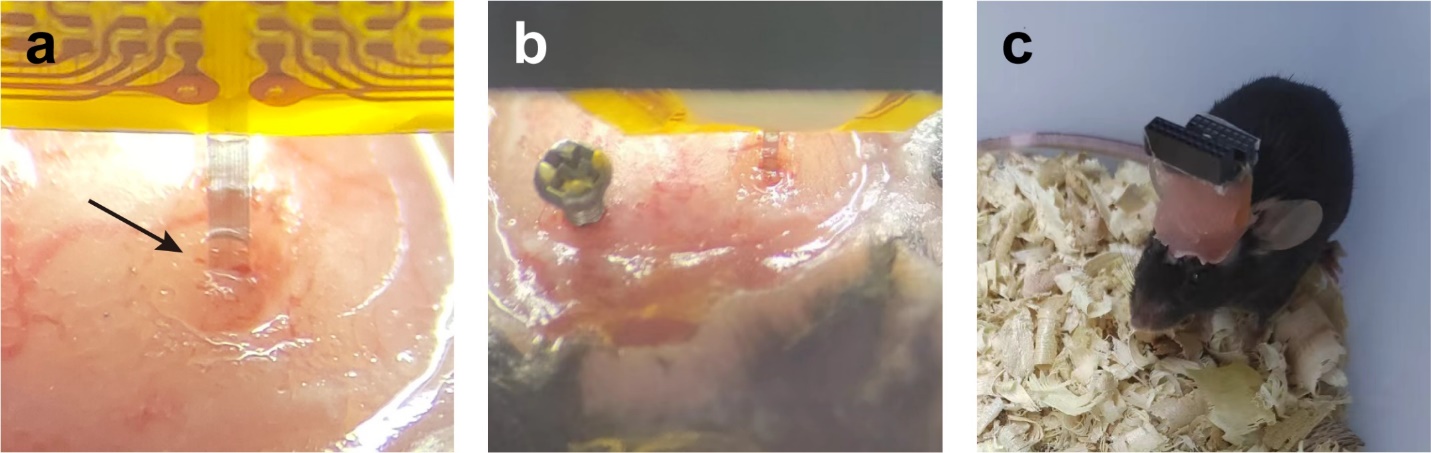


**Figure S3. Implantation of flexible electrode probe. (a)** A window is opened on the skull of the mouse, and a flexible array electrode is implanted into the brain. **(b)** Ground screws are fixed onto the skull of the mouse. **c** Flexible electrodes are secured onto the top of the mouse skull using dental cement.


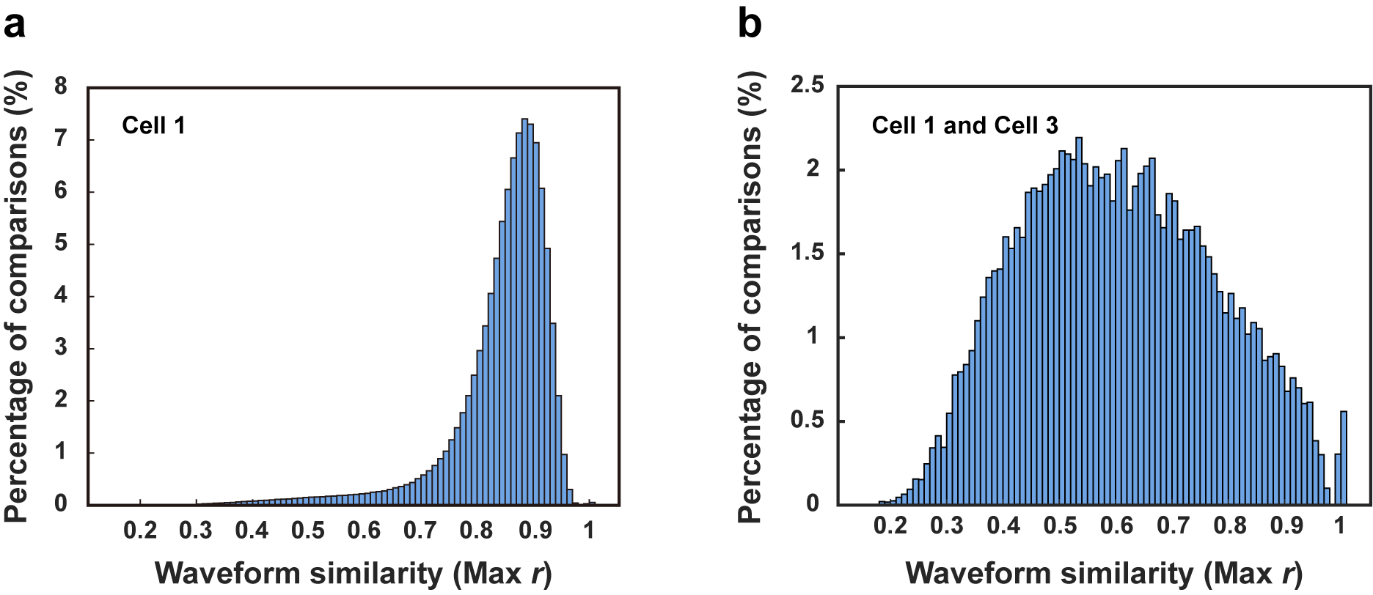


**Figure S4.** **Similarity between spike shapes for the same neuron over different months and for different neurons. (a)** The similarity of firing patterns of Cell1 over an eight-month period; 80.12% of the spike waveforms had a similarity coefficient (r) greater than 0.8. **(b)** The similarity of firing patterns between Cell1 and Cell3; only 15.22% of the similarity coefficients (r) exceeded 0.8, indicating that waveforms of different neurons typically differ significantly.


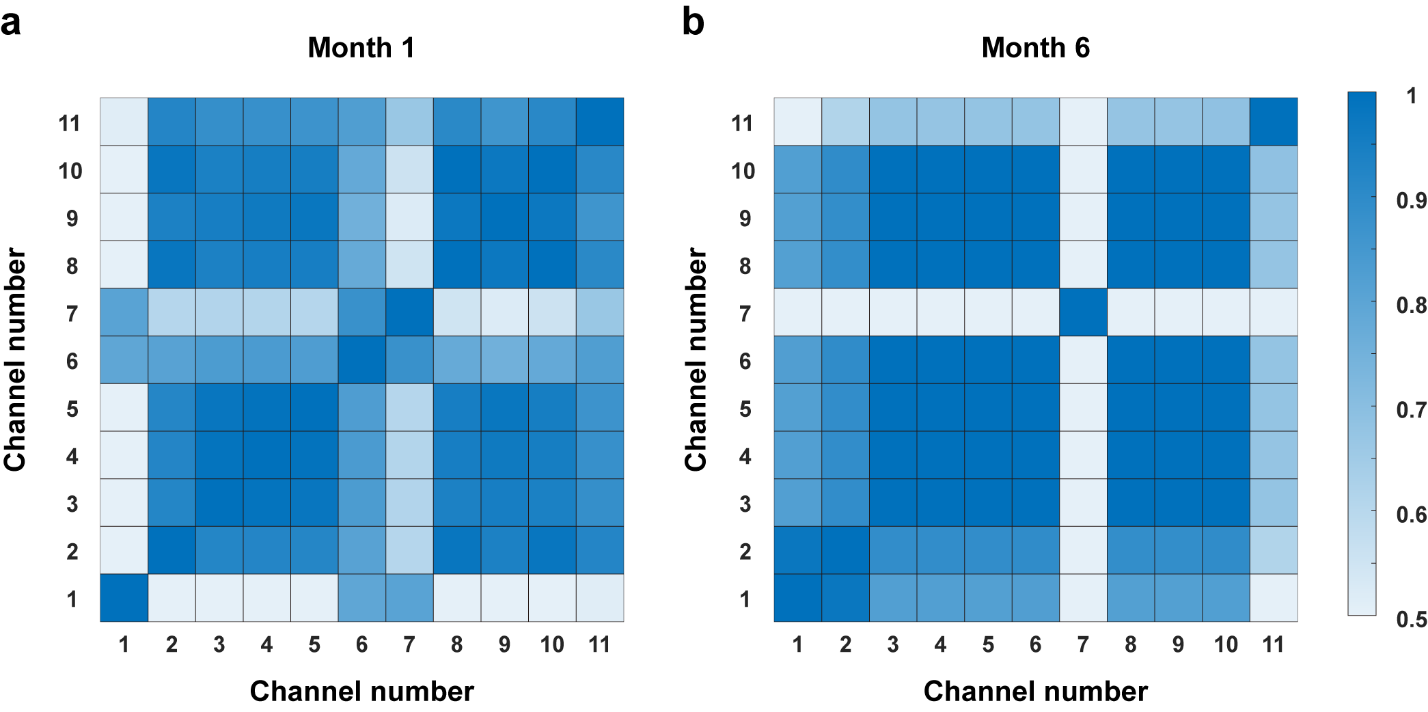


**Figure S5.** **Correlation maps of chronic LFP recordings.** **(a, b)** Correlation maps of 11-channel LFP recordings at one month (a) and six months (b) post-implantation. The color bar on the right displays the correlation coefficient between any two given channels. All heatmaps were calculated from 1-second-long data traces.


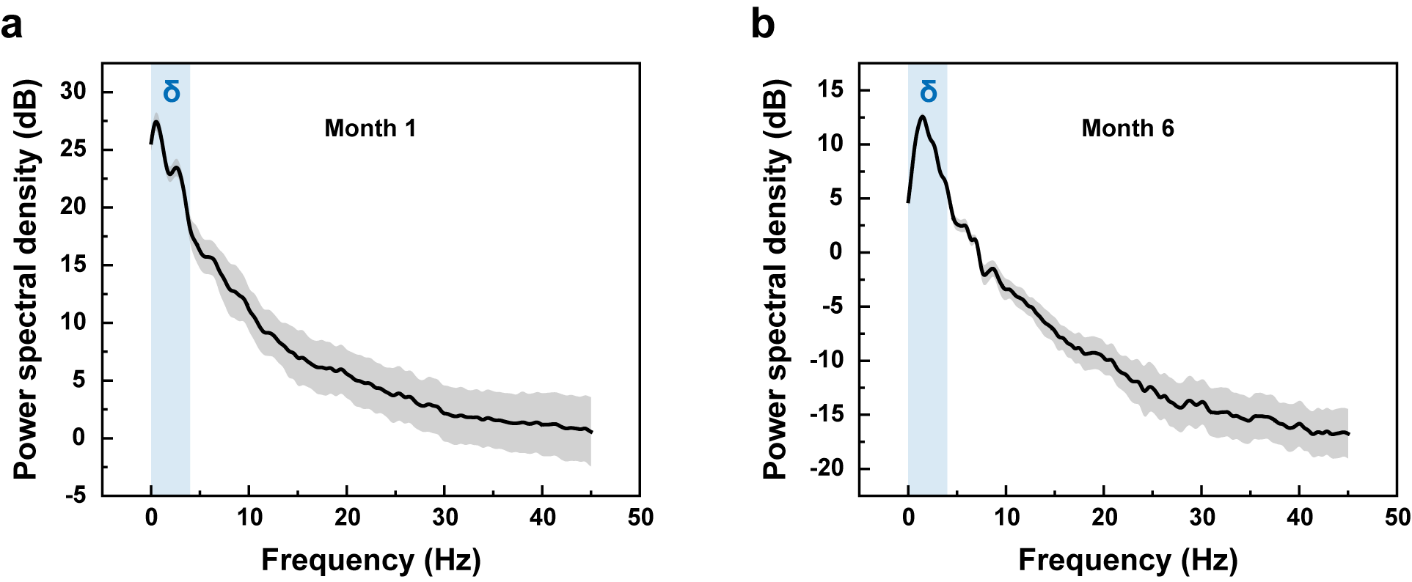


**Figure S6. Power spectral density of LFP.** **(a, b)**LFP power spectra from the first and sixth months. The frequency distribution trends of both are basically consistent, with peak frequencies concentrated in the delta (δ) band.


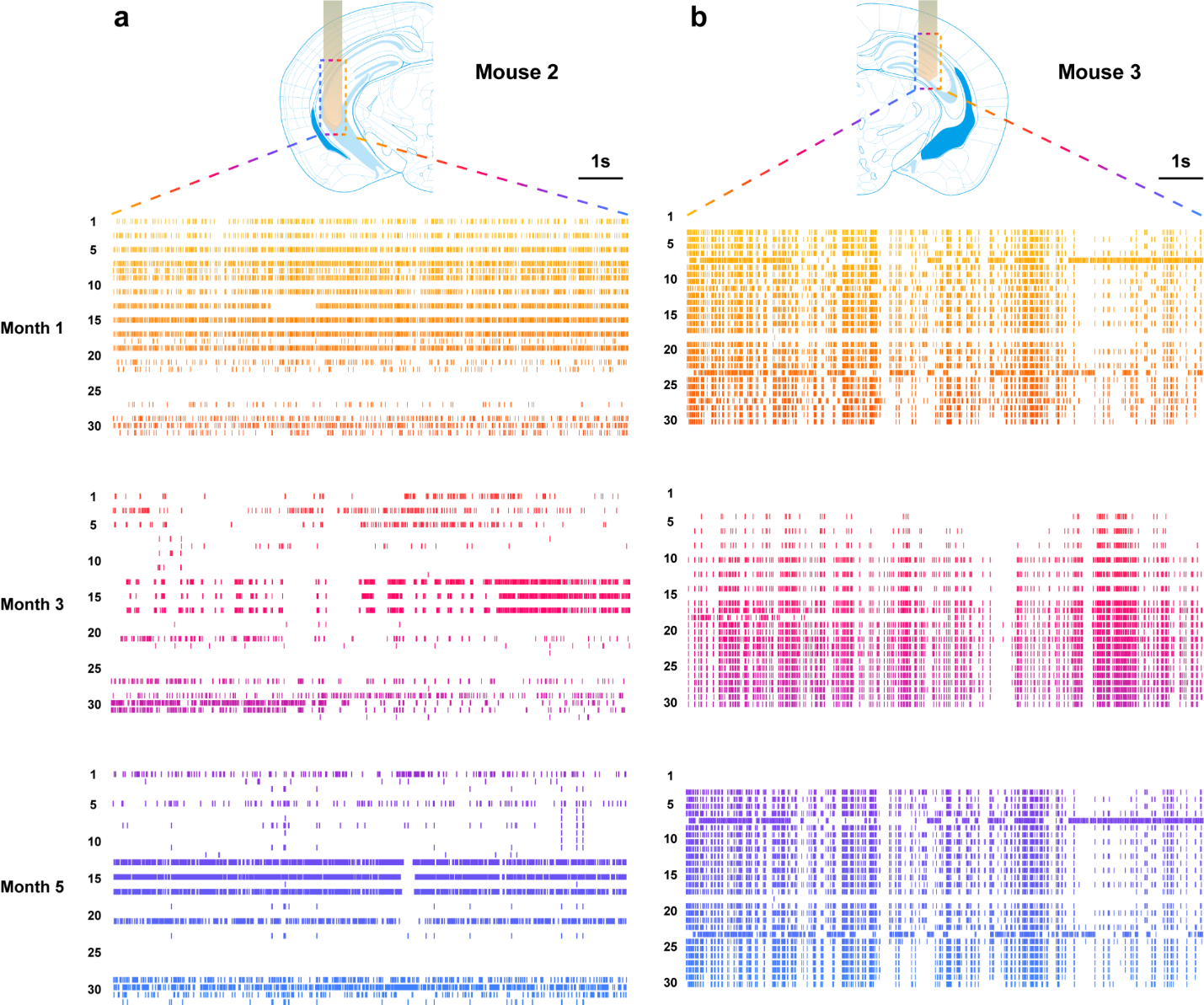


**Figure S7.** **Long-term *in vivo* recording.** Long-term recording data obtained from flexible probes implanted in the ventral and dorsal hippocampal CA1 and CA3 subregions of the left brain (mouse 2) and the dorsal hippocampal CA1 and CA3 areas of the right brain (mouse 3). The top row displays the electrode implantation sites, while the three rows below show neural firing data recorded at different times post-implantation.


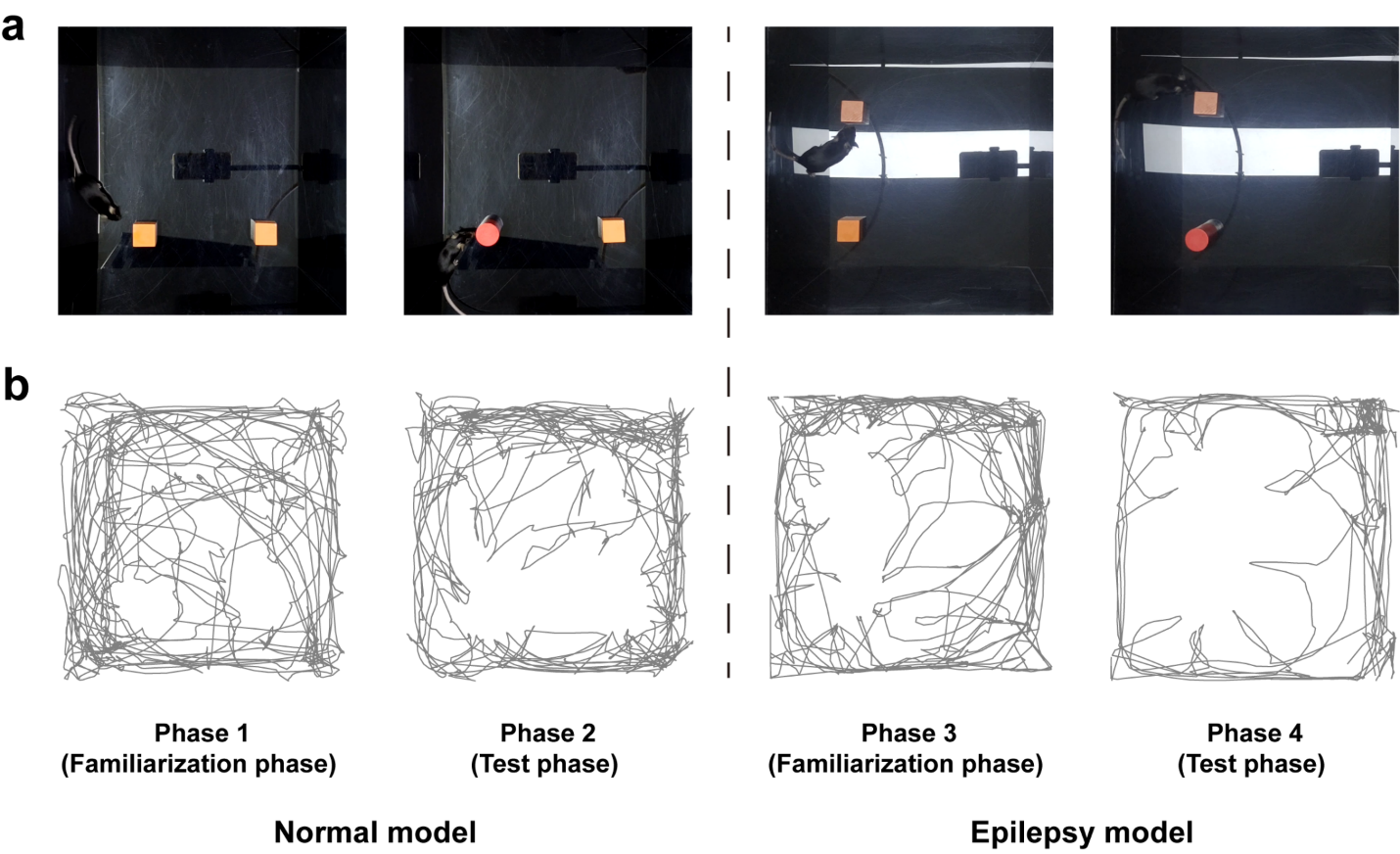


**Figure S8.** **Photographs and typical movement trajectories from the NOR test.** **(a)** Photographs of mice freely exploring objects during four NOR trials. **(b)** Typical exploration trajectories of mice before and after the onset of epilepsy across the four trials.


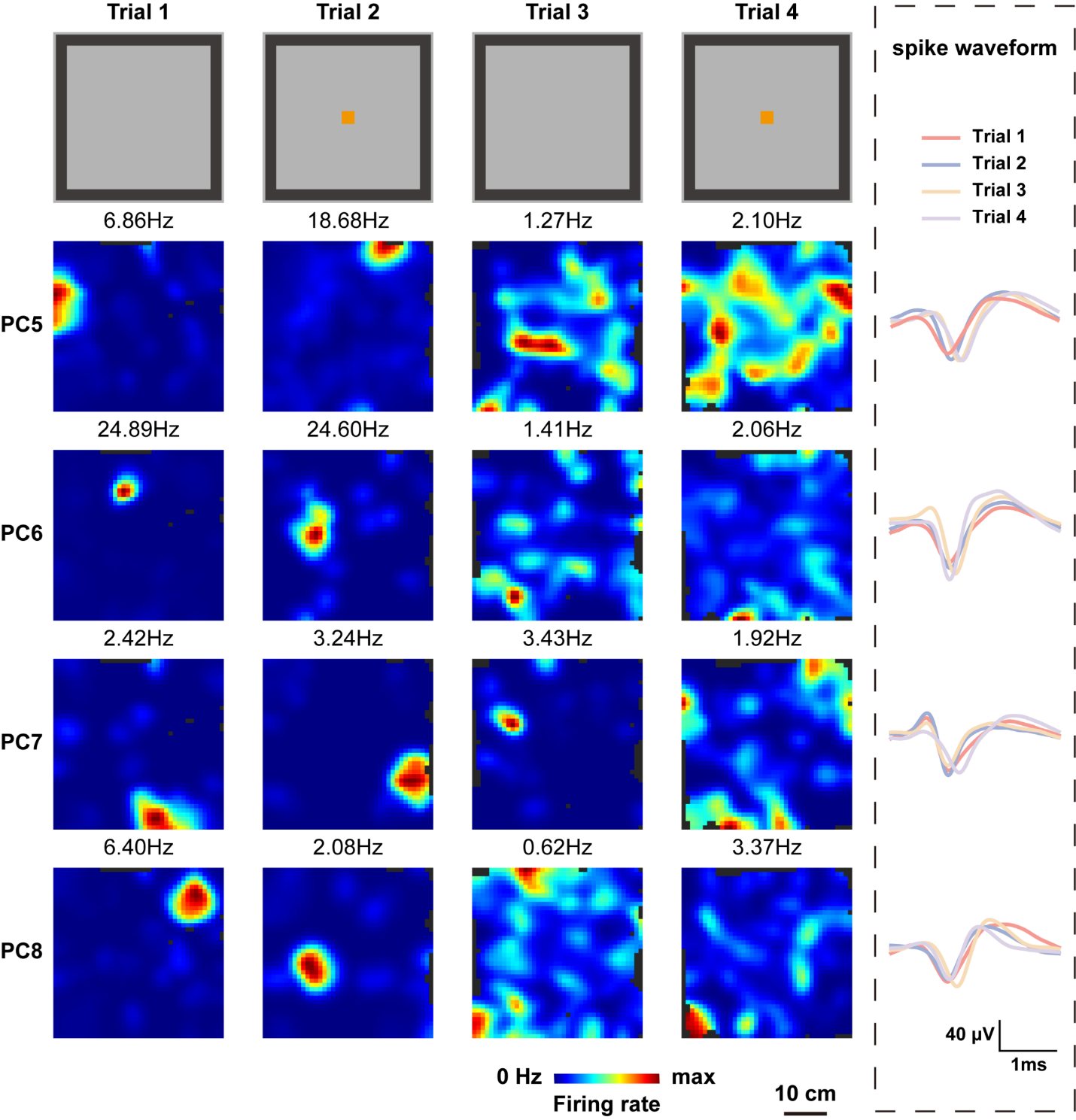


**Figure S9.** **Discharge of PC5-PC8 during open field exploration task.** The left four columns display the discharge heatmaps of PC5-PC8 in four exploration trials, with each row representing one cell (identified by the ID on the left). The color scale is normalized to the maximum peak rate, with the maximum peak rate of each trial indicated (above the text). The color scale is shown in the bottom right corner. The right side shows the spike discharge waveform changes of PC5-PC8 in the four trials.


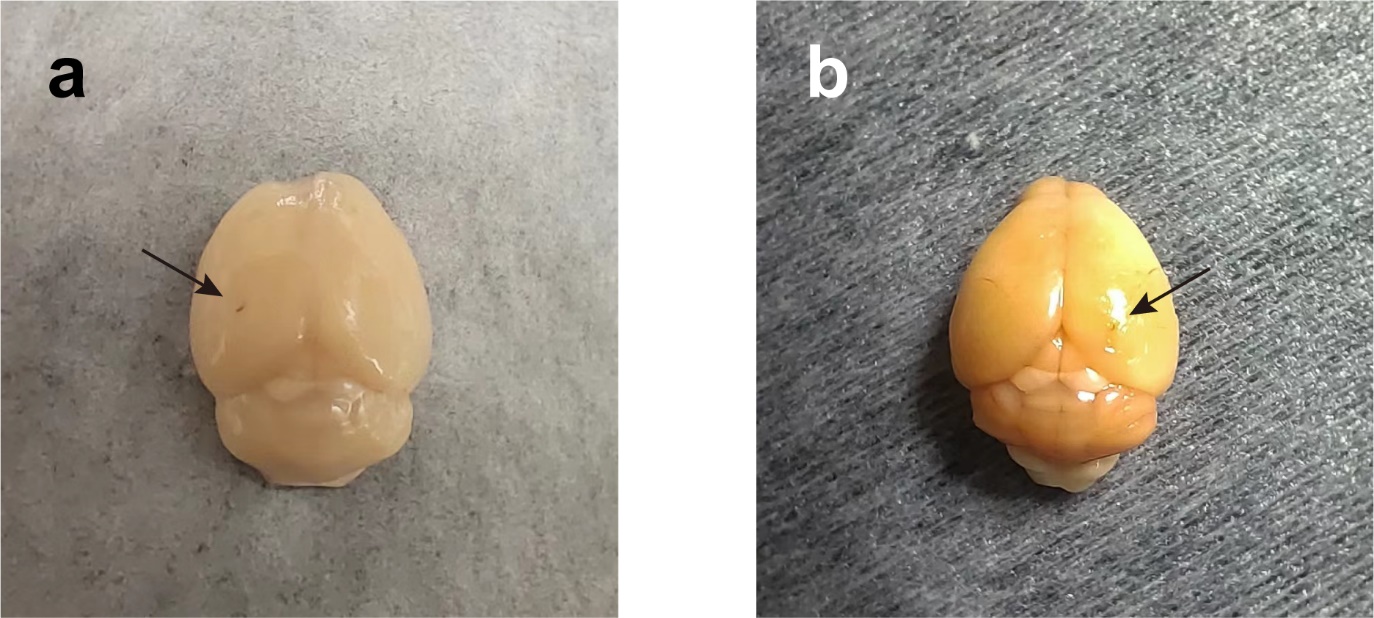


**Figure S10. Complete brain tissue obtained post-perfusion. (a, b)** Traces left by the electrodes implanted in the left and right brain hemispheres, respectively.

Supplementary Table

**Table S1. Dissolution rates of PEG with varying molecular weights.**

| Reference | PEG Molecular Weight | Dissolution time |
| --- | --- | --- |
| [10] | 1300 | 40 s |
| This work | 2000 | 2 min |
| [11] | 4000 | 200 s |
| [12] | 6000 | 6 min |
| [12] | 10000 | 15 min |
| [12] | 20000 | 30 min |

References

1. Han M, Wang Y, Jing L *et al.* Utilizing GO/PEDOT:PSS/PtNPs-enhanced high-stability microelectrode arrays for investigating epilepsy-induced striatal electrophysiology alterations. *Front Bioeng Biotechnol* 2024;**12**:1376151.

2. Jing L, Xu Z, Fan P *et al.* Deep brain implantable microelectrode arrays for detection and functional localization of the subthalamic nucleus in rats with Parkinson’s disease. *Bio-Design and Manufacturing* 2024, DOI: 10.1007/s42242-023-00266-y.

3. Kim D-H, Viventi J, Amsden JJ *et al.* Dissolvable films of silk fibroin for ultrathin conformal bio-integrated electronics. *Nature Mater* 2010;**9**:511–7.

4. Rockwood DN, Preda RC, Yücel T *et al.* Materials fabrication from Bombyx mori silk fibroin. *Nat Protoc* 2011;**6**:1612–31.

5. Curia G, Longo D, Biagini G *et al.* The pilocarpine model of temporal lobe epilepsy. *J Neurosci Methods* 2008;**172**:143–57.

6. Antunes M, Biala G. The novel object recognition memory: Neurobiology, test procedure, and its modifications. *Cogn Process* 2012;**13**:93–110.

7. Lu L, Igarashi KM, Witter MP *et al.* Topography of place maps along the CA3-to-CA2 axis of the hippocampus. *Neuron* 2015;**87**:1078–92.

8. Shuman T, Aharoni D, Cai DJ *et al.* Breakdown of spatial coding and interneuron synchronization in epileptic mice. *Nat Neurosci* 2020;**23**:229–38.

9. Skaggs WE, McNaughton BL, Gothard KM. An information-theoretic approach to deciphering the hippocampal code. 1992.

10. Barz F, Ruther P, Takeuchi S *et al.* Flexible silicon-polymer neural probe rigidified by dissolvable insertion vehicle for high-resolution neural recording with improved duration. *2015 28th IEEE International Conference on Micro Electro Mechanical Systems (MEMS)*. Estoril, Portugal: IEEE, 2015, 636–9.

11. Takeuchi S, Ziegler D, Yoshida Y *et al.* Parylene flexible neural probes integrated with microfluidic channels. *Lab Chip* 2005;**5**:519.

12. Felix S, Shah K, George D *et al.* Removable silicon insertion stiffeners for neural probes using polyethylene glycol as a biodissolvable adhesive. *2012 Annual International Conference of the IEEE Engineering in Medicine and Biology Society*. San Diego, CA: IEEE, 2012, 871–4.
